# Changing Pattern of Clinical Epidemiology on Hepatitis C Virus Infection in Southwest China

**DOI:** 10.5812/hepatmon.857

**Published:** 2012-03-28

**Authors:** Zehui Yan, Ke Fan, Yuming Wang, Yi Fan, Zhaoxia Tan, Guohong Deng

**Affiliations:** 1 Institute of Infectious Diseases, Southwest Hospital, the Third Military Medical University, Chongqing, China

**Keywords:** Epidemiology, Genotype, Hepatitis C

## Abstract

**Background:**

The changing pattern of hepatitis C virus (HCV) infection could have a significant impact on future medical prevention practices and therapies.

**Objectives:**

The purpose of this study was to describe the changing pattern of HCV infection in southwest China using clinical epidemiology, and to assess the association between the genotypes distribution and certain potential risk factors.

**Patients and Methods:**

A retrospective analysis which included 1208 subjects with chronic HCV registered at the Southwest Hospital (Chongqing, Southwest China) was performed. The information was reviewed and the data collected from clinical records and short telephone interviews when necessary. HCV genotypes were determined by nucleotide sequencing of the CE1 regions followed by phylogenic analysis with the published HCV genotype. HCV genotype distribution was analyzed according to the patients' age, gender, risk exposure, and the initial risk exposure.

**Results:**

Among the 1 208 patients, the HCV subtype 1b was the most prevalent (32.9%), followed by subtype 3b (18.9%), 6a (18.0%), 3a (12.8%) and 2a (10.4%), while subtypes 1a and 6k accounted for cases of HCV infection in only 9 and 3 cases respectively. Individuals older than 40 years were mainly infected with subtypes 1b and 2a, whereas younger patients were predominantly infected with genotypes 3 and 6. Subtypes 1b and 2a were observed more frequently among 44.4% and 16.0% patients respectively, with a history of invasive operations. Subtypes 3b and 6a constituted the majority of HCV infections among intravenous drug users (IDUs) (28.7% and 34.9%, respectively). A significant difference (P < 0.001) was observed between the HCV genotype distributions, according to the potential route of infection.

**Conclusion:**

In southwest China, the most common remaining subtype is the 1b genotype, but this has declined significantly among young patients. This is followed by subtype 3b and 6a which has increased significantly, especially among young patients. The distribution of such genotypes was also variable according to gender and age. The changing pattern of HCV infection was associated with changes in the modes of HCV acquisition, which raises an alarm signal concerning the major steps that need to be taken in order to reduce such infections in southwest China.

## 1. Background

The hepatitis C virus (HCV) is a major cause of liver disease worldwide and will be a potential source of substantial cases of morbidity and mortality in the future [1]. It is estimated that approximately 130-210 million individuals, i.e. 3% of the world's population, are chronically infected with the HCV [2]. The prevalence varies markedly from one geographical area to another and within the population assessed [3]. In Western Europe, HCV prevalence rates range from 0.4% to 3%. It is higher in Eastern Europe and the Middle East, in countries such as Egypt (15%), Romania (6%), Pakistan (4.7%) and in Ukraine (4.0%) [4][5]. There is a wide range of prevalence estimates among developing countries, and generally less data available to validate assumptions about the burden of disease, than in the developed world [6][7]. Studying the epidemiology of HCV infections plays an important role in the methods of its prevention [8][9]. HCV genotypes/subtypes are clinically very important as different genotypes are relevant to epidemiological questions, vaccine development, and the clinical management of chronic HCV infection [10][11]. HCV genotypes have also been showed to have unique patterns of geographic distribution and to be a major tool in HCV epidemiological studies [12]. HCV genotypes 1, 2 and 3 are commonly distributed all over the world and their relative prevalence varies from one geographic area to another, while genotypes 4, 5, and 6 are generally found only in certain areas. For example, HCV subtypes 1a and 1b are the most common genotypes in the United States [13], Europe [14][15][16][17] and Japan [18]. HCV-3 (predominantly 3a) is the most prevalent genotype circulating in India [19] and Pakistan [20]. HCV genotype 4 appears to be more prevalent in North Africa [21] and the Middle East [22]. HCV genotypes 5 and 6 seem to be confined to South Africa [23] and Hong Kong [24], respectively. The distribution of HCV genotypes varies significantly according to changing modes of HCV acquisition [25]. Therefore, the epidemiological studies of HCV genotypes in different risk populations may provide a better understanding into the nature of HCV infection and its spread. The epidemiological patterns of HCV vary greatly among the different countries and even among the regions of the same country. The epidemiology varies geographically and temporally due to its distribution and the evolution of risk factors [26]. The epidemiology of HCV is highly associated with certain risk groups such as: recipients of blood and/or blood products, injecting drug use (IDU), sexual transmission, inadequate sterilizing of medical equipment, body piercing and the sharing of razors and other personal items which are contaminated with HCV [20]. With the implementation of mandatory HCV screening of blood and blood products in the early 1990s, the number of post-transfusion infections had already decreased dramatically, while IDU has accelerated in China [27][28][29]. In China, previous studies have indicated that the most prevalent HCV subtype was 1b, followed by 2a [30][31], while recently studies have shown new trends of HCV infection in some regions of China [32][33]. Thus, it is important to investigate the clinical epidemiological shift in the prevalence of the predominant HCV genotype and the significance of the changes.

## 2. Objectives

An epidemiological study concerning HCV genotypic distribution in the southwest region of China has been reported in a small population in this region [34]. However, studies on the changing pattern of clinical epidemiology on hepatitis C virus infections in southwest China are scarce and the HCV genotype distribution in this region needs to be determined in larger sample studies. The objectives of this study were to describe the clinical epidemiological changing patterns of HCV infection in southwest China, and to determine whether there are any associations between HCV genotypes and the mode of acquisition, and how this could assist in the prevention of such virus infections among the people of southwest China.

## 3. Patients and Methods

### 3.1. Study Participants

Individuals with HCV infection who were registered and followed up at the Southwest Hospital (Chongqing,Southwest China) from January 2004 and June 2011 were included for the primary selection. All patients finally enrolled in the study had anti-HCV positivity and detectable serum HCV RNA for at least 6 months.Information on these patients were obtained mainly from clinical records retrospectively and short telephone interviews with a structured questionnaire when necessary. The study was designed to collect the data and extract information from each patient including; age, gender, year of diagnosis and risk factors for HCV. The risk exposures considered in this study were; transfusion of blood or blood products, blood plasma donation, hemodialysis, tattoos, acupuncture therapy, injecting drug use (IDU), high-risk sexual practices, occupational exposure, surgical operations, intra-family transmission, etc. Those patients who denied any risk factors were assigned to a group labeled unknown. Patients with more than one risk factor were assigned to a single risk group according to the hierarchy described above. There was prospective informed consent and interviews as well as prospective HCV genotyping in this retrospective study. All subjects provided informed consent to participate in the study, as approved by the ethical committee of the Southwest Hospital, Chongqing, China.

### 3.2. Laboratory and Clinical Evaluation of HCV Infection

Serum samples were collected from the patients and were tested positive for HCV antibody using the 3rd generation commercial Enzyme Linked Immunosorbent Assay Kits, INNO-LIA HCV Ab III update Immunoassay (LIA, Innogenetics S.r.l., Gent, Belgium). Such an immunoassay is known to have a high specificity and sensitivity (over 99%), with minimal or no limitation in detecting the HCV antibody. The HCV RNA load was tested on the seroreactive samples using quantitative real-time PCR (ABI Prism 7000, Applied Biosystems, Foster City, CA). The diagnostic criteria for the HCV were based on the combination of; clinical history, physical examination, imaging and laboratory data and/or histology. Diagnosis of cirrhosis was made by positive histological findings or an elevated clinical/laboratory data combined with at least one positive image (ultrasonography, computed tomography, magnetic resonance imaging and/or fibroscan).

### 3.3. HCV Genotyping

HCV RNA was extracted using the commercial extraction kit QIAamp Viral RNA Mini Kits (Qiagen,Shanghai, China). Extracted RNA was used for reverse transcription (RT) followed by nested PCR with two sets of conserved primers deduced from the core-envelope 1 (CE1) region of the HCV genome as described in previous research [35]. Both the RT and nested PCR were performed in a MyCycler Thermal Cycler (BIO-RAD, Hercules, CA, USA). The amplicon fragment was 474bp in length. Genotypes of all of the samples were determined by nucleotide sequencing of the CE1 region followed by phylogenic analysis with

the published HCV genotype references in GenBank. Electrophoresis of the sequenced products and analysis of the electrophoregrams were performed with the use of sequenator DNA ABI Prism 3730 (Applied Biosystems, Foster City, CA). Phylogenic comparison to reference sequences of particular subtypes was performed with the Viral Genotyping Tool on the NCBI website.

### 3.4. Statistical Analysis

Statistical analysis was performed using SPSS software (version 9.0; SPSS Inc, Chicago, IL). A 2-sided pvalue less than 0.05 was considered to be significant. χ2 tests were performed to examine the differences in the distribution of categorical variables. Quantitative variables were expressed as mean ± standard deviation and were compared by a Student's t-test. Differences in the proportion of qualitative variables were tested with non-parametric tests, Yates correlation. A multivariate analysis was conducted using logistic regression in order to verify which variables statistically had an influence on the HCV infection.

## 4. Results

### 4.1. Clinical Epidemiological Characteristics of Study Population

A total of 1 208 patients were enrolled in this study from different geographical provinces in southwest China. All patients are native residents of southwest China and the majority of them were from Chongqing (n = 745; 61.7%), followed by Sichuan (n = 256; 21.2%) and Guizhou (n = 169; 13.9%), while the others came from other southwest provinces (Yunnan, Guangxi and Xizang). The ages of the patients in the study ranged from 16 to 88 years with a mean age (±SD) of 38.5 ± 11.5 years. Of these, 707 patients (58.5%) were male and 501 patients (41.5%) were female. Out of a total of 1 208 cases, the HCV genotype was available for 1 137 patients (94.1%) and non-detectable in 71 subjects (5.9%). Genotypes 1, 2, 3, and 6 were found in our population. HCV subtypes 1b was the most prevalent subtype (n = 398, 32.9%), followed by subtype 3b (n = 228, 18.9%), 6a (n = 218, 18.0%), 3a (n = 155, 12.8%) and 2a (n = 126, 10.4%), while subtype 1a and 6k accounted for HCV infection in only 9 (0.7%) and 3 (0.3%) individuals respec ively. No differences were found in serum HCV-RNA levels among the HCV genotypes; mean levels of HCV-RNA (copies/ml) in log10 were: 5.9 ± 1.4 for genotype 1, 6.2 ± 0.8 for genotype 2, and 5.7 ± 0.6 for genotype 3, and 6.4 ± 1.5 for genotype 6. Furthermore, the main HCV genotype distribution differed significantly by gender. Subtype 3a (men/women: 15.9% vs. 7.8%, P = 0.0003), 3b (21.6% vs. 14.4%, P = 0.009) and 6a (20.3% vs. 14.37%, P = 0.03) were more frequent in men than in women. In contrast, subtype 1b (men/women: 29.11% vs. 39.22%, P = 0.011) and 2a (8.013% vs. 14.4%, P = 0.002) were more frequent in women than in men. Although efforts were made to obtain the potential route of infection for each patient in our study, the mode of HCV acquisition in 210 (17.4%) cases remained unknown. Of the 1 208 patients, 406 (33.6%) had a history of transfusion of blood and/or blood products, 352 (29.4%) had a history of IDU, 144 (11.9%) had a history of invasive operation (hemodialysis, tattoos, acupuncture therapy, de ntal procedures, occupational exposure, etc); 96 (7.9%) had a history of high-risk sexual practices (history of promiscuity, infected from family members). No significant differences were observed between the genders of the sex-related (P = 0.364) and sourceunknown chronic hepatitis C (P = 0.228). However, there were more frequent cases in women than in men (P ≤ 0.001) for transfusion-related (men/women: 29.24% vs. 40.74%, P = 0.004) and operation-related chronic hepatitis C (CHC) (men/women: 9.48% vs. 16.91%, P = 0.003). In contrast, IDU's were more frequently male than female (men/women: 36.58% vs. 16.99%, P = 0.003).

Of the 1 208 patients, 873 had been examined by at least one kind of image examination or with a histological biopsy at the time of their diagnosis, while 335 patients lacked adequate information to diagnose or exclude cirrhosis. Table 1 lists the demographic characteristics and genotype distribution in the 873 chronic HCV patients with and without cirrhosis. Based on positive histological findings, or an elevated clinical/laboratory data combined with at least one positive image, evidence of obvious or extensive fibrosis was confirmed in 276 (31.7%) individuals.. Between patients with and without cirrhosis, statistically significant differences were observed with respect to age (P < 0.001), sex (P = 0.011), route of transmission (P < 0.001) and HCV genotype distribution (P = 0.029). Cirrhosis was present in a large number of blood-transmission-related patients (41.8%) (P < 0.001) and only in a significantly smaller proportion of IDU patients (15.9%) (P < 0.001). Cirrhosis was also present in operation-transmission-related patients (28.3%) and sex-transmission-related patients (36.2%), however, statistically significant differences were not observed between patients with or without cirrhosis for these two possible routes of transmission. With respect to the HCV subtype distribution, cirrhosis was present in a significantly large proportion of patients with subtype 1b (P < 0.001), while for the other subtypes, no significant difference was observed between patients with or without cirrhosis (P > 0.05). In a multivariate analysis, cirrhosis was found to be associated only with the age of the patients (P < 0.001).

**Table 1:** Demographic Characteristics and Genotype Distribution in Chronic HCV Patients with and without Cirrhosis

	**Cirrhosis**	**Non-cirrhosis**	**P value a**
Patients, No. (%)^b^	276 (31.7)	597 (68.3)	
Age^c^,y,mean ± SD	55.8 ± 11.3	41.6 ± 12.4	< 0.00
Gender			0.011
Male, No. (%)	133 (48.2)	343 (57.5)	0.601
Female, No. (%)	143 (51.8)	254 (42.5)	
Alcohol drinkers d, No. (%)	37 (13.4)	88 (14.7)	
Route of transmission e, No. (%)			< 0.001^f^
Blood	123 (44.6)	171 (28.6)	< 0.001
Operation	32 (11.6)	81 (13.6)	< 0.419
IDU	43 (15.6)	228 (38.2)	< 0.001
SEX	21(7.6)	37(6.2)	0.436
Unknown	57(20.6)	80(13.4)	0.007
Genotype distribution, No. (%)			0.029 g
1a	-	1 (1.7)	< 0.001
1a	135 (48.9)	202 (33.8)	0.383
2a	26 (9.4)	68 (11.4)	0.077
3a	29 (10.5.)	89 (16.2)	0.092
3b	44 (15.9)	124 (20.7)	0.116
6a	34 (12.3)	98 (16.4)	
Non-detectable h	8 (2.9)	15 (2.5)	

^a^ P values were given for the comparison between cirrhosis and non-cirrhosis groups by χ^2^ tests.

^b^ Out of all of the 1 208 patients, 873 had been examined by at least one kind of image method or with histological biopsy at the time of diagnosis. Based on positive histological findings or an elevated clinical/laboratory data level, combined with at least one positive image result, 276 individuals had evidence of obvious cirrhosis or extensive fibrosis.

^c^ In a multivariate analysis, the age of the patients was the only factor associated with the presence of cirrhosis (P < 0.001).

^d^ Drinker, was defined as alcohol consumption of ≥ 40 g/week for men and ≥ 20 g/week for women, which included occasional drinkers and daily drinkers.

^e^ Blood, patients with a history of transfusion blood or blood products; Operation, patients with a history of invasive operation; IDU, patients with a history of IDU; Sex, patients with a history of promiscuity and unsafe sex; Unknown, patients where the source of infection was unclear and unknown.

^f^ P values were given by 2×5 table chi-square between cirrhosis and non-cirrhosis groups.

^g^ P values were given by 2×7 table chi-square between cirrhosis and non-cirrhosis groups.

^h^ Non-detectable, patients whose HCV genotype could not be identified.

### 4.2. Time Changes of Genotypes Distribution and Routes of Transmission

**Figure 1:**
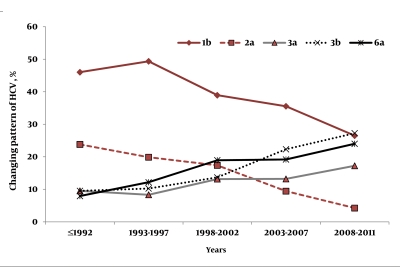
Changing Pattern of HCV Genotypes during the Past 20 Years

**Figure 2:**
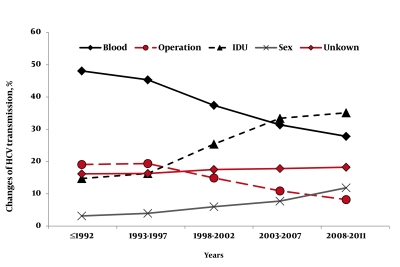
Changes of Possible Mode of HCV Transmission during the Past 20 Years

According to the year of HCV infection, we classified the whole population into five subgroups and a gradual upward trend of patients with chronic hepatitis C (CHC) was observed consistent with the time of disease acquisition. The number of patients in this hospital-based population was 786 (65.1%) over the previous ten years (2002-2011), while only 353 patients (29.2%) were diagnosed in the years from 1992-2001. As Figure 1 showed, genotype 1b remained the predominant genotype throughout the over 20 year study period. The proportions of genotypes 2a and 3a remained stable throughout this time. A sharp reduction in the proportion of subjects with genotypes 1 and a sharp increase in the proportion of subjects with genotype 3b and 6a was observed over this period, and statistical analysis was performed to identify the associations between the genotypes and time of infection (year) if any. The subtypes 3a and 2a were observed to have a statistically non-significant correlation with time (years) (P > 0.05), while the HCV subtypes 1b, 3b and 6a showed significantly higher correlations with years (P < 0.05). Similarly, we also observed the changing pattern of the sources of infection during the last 20 years in the study population. As Figure 2 shows, a history of transfusion of blood and/or blood products and a history of IDU were the main sources of infection. The proportion of unknown sources of infection has remained stable throughout the study period. For the sex-related CHC, no significant correlation with time (years) (P > 0.05) was observed, however, an increase in this trend could be seen with years. A significant association between the source of infection and the time of the infection (year) was observed. A significant reduction in transfusion-related and operation-related CHC was observed over time. On the contrary there was a dramatic increase in IDU related hepatitis C, mainly after 1997 (P ≤ 0.001).

### 4.3. Association of genotypes with the risk factors

**Figure 3:**
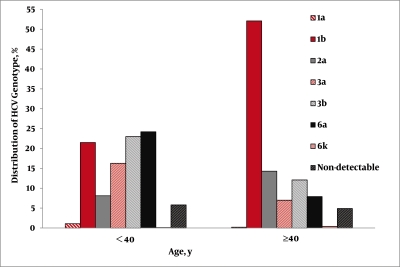
Distribution of HCV Genotype in the Subgroups of > 40 years and < 40 years

Table 2 translates the age wise distribution of the different genotypes observed in the current study. The highest percentages (36.9%) were in the age group 31–40 years. The second most abundant age group of patients with all genotypes was in the 41–50 years age group with the occurrence of 21.3% followed by the 21–30 years group with 20.2%, 10.9% in the 51–60 years age group, 5.7% in the ≥ 61 years age group and 5.2% in ≤ 20 years age group. A notable variation in the distribution of HCV subtypes in the different age groups was observed (P < 0.001). Subtypes 3a, 3b, and 6a were detected in 22.9%, 22.9%, and 20.9% of patients aged 21–30 years, respectively. On the other hand, subtypes 1b and 2a were present in only 18.9% and 8.62% of cases respectively in the same age group. In individuals 31–40 years old, subtypes 3b (24.2%), and 6a (27.1%) were also statistically more prevalent than in genotype subtypes 1b (19.9%), 2a (14.2%) and 3a (8.7%). In the next group (41–50 years old), HCV subtypes 1b, 2a and 3b were found predominantly (46.9%, 14.2% and 14.2%, respectively), while patients with subtypes 3a, and 6a were relatively rare (8.7%). In the last two groups (51–60 and older than 60 years), subtypes 1b and 2a were statistically predominant, accounting respectively for 73.5% and 72.5% of all infections observed. Among patients with a genotype which could not be identified, the frequency of the different age groups fluctuated between the 4-7% range and no significant difference was found. Figure 3 also indicates that subtype 1b (21.38% vs. 52.09%) and 2a (8.11% vs. 14.29%) were less common in patients <40 years of age, than in those ≥ 40 years old (P < 0.05), whereas, in genotype 3a (16.03 % vs. 7.03%), 3b (22.97% vs. 12.09%) and 6a (24.17% vs. 7.91%) these were much more common in patients < 40 years old than in those ≥ 40 years old and genotype 6a was less common in patients ≥ 40 years old (P < 0.001). Table 3 depicts the distribution of the HCV genotype/subtype in the different risk groups. A significant difference (P < 0.001) in the HCV genotype distribution, according to the potential route of infection, was observed. Subtype 1b was found more frequently among 51.2% patients with a history of blood transfusion and 44.4% of patients with a history of invasive operation and medical procedure，while this occurred significantly less frequently in the IDU group (15.3%). Additionally, subtypes 1b and 2a were also predominantly observed more frequently among 44.4% and 16.0% patients respectively with a history of invasive operation. In contrast, subtypes 3b and 6a constituted the majority of HCV infections in drug users (28.7% and 34.9%, respectively), occurring significantly more frequently than HCV infection caused by a transfusion of blood or blood products (P < 0.05). Subtypes 3b (34.4%) and 1b (32.3%) were observed more often in patients with a history of promiscuity and unsafe sex than in other subtypes with this source of infection. Subtype 3a, 1b and the non-detectable group were found in 31.9%, 19.5 and 19.1% in patients with unknown sources of infection, respectively.

**Table 2:** HCV Genotypes and Risk Factors Distribution among 1 208 Chronic Hepatitis C Patients According to Age Group

**Genotype**	**Age Group, No. (%)**						**Total, No.**
	**≤ 20, y (n = 63)**	**21-30, y (n = 244)**	**31-40, y (n = 446)**	**41-50, y (n = 257)**	**51-60, y (n = 132)**	**≥ 61, y (n = 69)**	
1a	0 (0)	2 (0.82)	6 (1.35)	1 (0.39)	0 (0)	0 (0)	9
1b	26 (41.3)	46 (18.9)	89 (19.9)	119 (46.9)	77 (58.3)	41 (59.4)	398
2a	4 (6.3)	21 (8.6)	36 (8.1)	36 (14.2)	20 (15.2)	9 (13.1)	126
3a	10 (15.9)	56 (22.9)	57 (12.8)	22 (8.7)	7 (5.3)	3 (4.35)	155
3b	9 (14.3)	56 (22.9)	108 (24.2)	36 (14.2)	12 (9.1)	7 (10.1)	228
6a	10 (15.9)	51 (20.9)	121 (27.1)	22 (8.7)	9 (6.8)	5 (7.25)	218
6k	-	-	1 (0.22)	1 (0.39)	1 (0.76)	-	3
Non-detectable a	4 (6.3)	12 (4.9)	28 (6.3)	17 (6.7)	6 (4.55)	4 (5.79)	71

^a^ Patients who had a HCV genotype that could not be identified.

**Table 3:** HCV Genotype Distribution According to Route of Infection

**Subtype (n)**	**Potential Route of Infection, No. (%)**					**Total, No.**
	**Blood a(n = 406)**	**Operation a(n = 144)**	**IDU a(n = 352)**	**Sex a(n = 96)**	**Unknown a(n = 210)**	
1a	4 (1.0)	1 (0.7)	1 (0.3)	1 (1.0)	2 (1.0)	9
1b	208 (51.2)	64 (44.4)	54 (15.3)	31 (32.3)	41 (19.5)	398
2a	67 (16.5)	23 (16.0)	12 (3.4)	5 (5.2)	19 (9.0)	126
3a	19 (4.7)	10 (6.9)	52 (14.7)	7 (7.3)	67 (31.9)	155
3b	48 (11.8)	22 (15.3)	101 (28.7)	33 (34.4)	24 (11.4)	228
6a	43 (10.6)	18 (12.5)	123 (34.9)	17 (17.7)	17 (8.1)	218
6k	3 (0.7)	-	-	-	-	3
Non-detectable a	14 (3.4)	6 (4.2)	9 (2.6)	2 (2.1)	40 (19.1)	71

^a^ Blood, patients with a history of transfusion blood or blood production; Operation, patients with a history of invasive operation; IDU, patients with a history of IDU; Sex, patients with a history of promiscuity and unsafe sex; Unknown, patients where the source of infection was unclear and unknown; Non-detectable, patients with a HCV genotype that could not be identified.

## 5. Discussion

In our study, we observed the clinical epidemiological characteristics and substantial changes in genotype distribution in a very large number of patients infected with chronic HCV infections over the last 20 years in southwest China. We also analyzed the association between the distribution of genotypes and certain potential risk factors. According to our data, the relative prevalence of genotypes 3a, 3b and 6a has increased significantly, and that of genotypes 1b and 2a has declined considerably in the last 20 years in southwest China. This shift in distribution was mainly correlated to changes in the mode of HCV transmission, gender and time of the HCV infection acquisition. To the best of our knowledge no other study is available from this region consisting of such a large number of patient’s HCV genotypes/subtypes and the changing patterns seen over time in these frequencies. The first important finding of the current study is the observation that the prevalence frequencies of HCV genotypes, particularly the subtypes, has changed over the last 20 years in southwest China. Unfortunately, the prevalence of HCV infections, especially, the prevalence of HCV infection with genotype 3 and 6, appears to show a rising trend. One of the possible reasons for this increase is an increase in the chances of contracting an infection with an increase in personnel exchanges. Another important reason for this increase is the enhanced awareness of patient care with the improvement of living standards, so, we are better equipped to detect these cases in our hospital-based population. One item of good news is that the frequency of the HCV subtype 1b decreased from 58.6% before 1992 to 25% in the previous five years, as the sub-genotype 1b is considered to be a poor-responder to anti-viral therapies when compared to other genotypes [3]. More good news is that the proportion of patients with genotype 2a has gradually decreased and after 2008 only a few cases with this genotype (4.3%) were seen and it has almost been eradicated from southwest China. This decrease in the frequency of sub-genotypes 1b and 2a is multifactorial. Firstly, these genotypes are mostly spread through the general route of; major/minor transfusion of blood and/or blood products, operations, surgeries and dental procedures. Due to awareness programs at the public level, sterilized surgical procedures and equipment are now used for these procedures and that may be an important reason in limiting its further spread. Secondly, a highly probable reason for the complete eradication of genotype 2a may be due to the high sustained virological response rates seen for this particular genotype [36]. The results of a systematic review of HCV epidemiology in Asia, Australia and Egypt corresponds well with our observations [7]. Studies from Europe have also shown a decrease in genotype1b and 2a and the emergence of genotype 3 [14][15][16]. In a previous study conducted in southwest China, although it evaluated the relative frequencies of HCV genotypes in only 367 unselected patients, it also showed a similar trend in genotype distribution [34].

The second interesting finding of the current study is the observation that the mode of HCV transmission has also changed in the last 20 years in southwest China. A history of blood and/or blood products transfusion remained the main source of infection throughout this period of time where the rate gradually decreased from 47.8% to 27.6% in this region. It is worth mentioning that the risk of transfusion-related HCV-hepatitis progressively declined, possibly due to the implementation of an all-volunteer blood donor system and the effective virus-inactivation procedures for blood derivatives after 1997. On the other hand there was a dramatic increase in IDU related hepatitis C, mainly after 1997 when its rate gradually increased from 14.5% to 29.1%. The key reason for this dramatic increase in IDU related hepatitis C may be due to the fact that most regions of southwest China are connected to southeast Asia which is one of the world’s most important opium planting and drug producing bases, and southwest China has been an important transit area for the smuggling of drugs to other regions of China [27]. The third finding is the association between the genotypes distribution and certain potential risk factors, such as gender and age of HCV infection acquisition. In this study, there was a variation of HCV genotypes among male and female patients. Subtypes 3a, 3b and 6a were found more frequently in men than in women, while subtypes 1b and 2a were more frequent in women than in men. Previous studies on the association of gender with specific HCV genotypes were found to be equivocal. In Luxembourg, the prevalence of HCV genotype 3 was found to be significantly associated with males while genotype 2 was more frequent in females [37]. However, no significant difference was found in Pakistan as the distributions of HCV genotypes were similar in both male and female patients [38][39]. Unlike a recent study [40], we were able to find an association between HCV genotypes/subtypes and age, where a predominant prevalence of HCV genotypes 3 and 6 was seen in younger age patients. However, it should be noted that age at HCV infection, although found to be associated with the changes in HCV genotypes’ distribution over time in univariable analysis, its significance did not persist in multivariable analysis. This is because, the younger HCV positive patients belonged to the group of IDU acquired infection, while the older ones belonged to the blood transfusion acquired infection group. These results are consistent with other reports from different geographical regions of the world [16][17][39][41][42].

Most importantly, a significant difference in HCV genotype distribution by the source of infection was observed. Subtype 1b was found more frequently among patients with a history of blood transfusion and invasive operation, while subtypes 3b and 6a were more frequent among patients infected via IDU. This observation might reflect a tendency for a gradual change in the spectrum of HCV variants circulating in the southwest China population. There are two possible explanations: the first is that there are significantly more IDUs among younger patients compared to the older group of patients, and genotypes 3 and 6 are more frequent among IDUs than in other patients. However, the altered genotype patterns reported here are also found among patients who only have a history of transfusion, or whose modes of HCV acquisition are unclear. Logistic regression analysis, with adjustment for gender and different modes of HCV acquisition, also demonstrated that the age of the infected subjects was a direct explanatory variable for HCV genotype distribution. Thus, the observed shift in HCV genotype distribution cannot be attributed exclusively to changes in the epidemiological relevance of different known risk factors for HCV transmission. The second possible reason is that those infected via IDU were infected with ‘imported’ genotypes that they then gradually transmitted to the non-IDU populations through blood donation or other routes. It can perhaps be presumed that the different distribution of HCV genotypes observed in the IDU population is due to an increase of “imported” genotypes [3][6]. Therefore, younger people have a greater chance of being infected with the “imported” genotypes than older people, which is in agreement with the significantly higher frequency of genotypes 3 and 6 among patients infected after 1997 than in those infected before 1997, as found in the present study. Furthermore, through the evaluation of patients with a known genotype and a defined duration of HCV infection, we have recorded a significant increase in the proportion of patients infected with genotype 3b and 6a mainly after the 2000’s and a decline in post-transfusion HCV infections over time. Therefore, blood safety and improvement in infection control practices have paved the way for IDU to become, not only the main risk factor for HCV transmission, but also to alter the genotype distribution among patients with HCV hepatitis. As a retrospective study performed only in one centre, some limitations are difficult to avoid. First, although our patients came from various provinces and municipalities in southwest China, our hospital-based population may not be representative of the general population in southwest China. Second, the retrospective design of the current study may have led to selection bias. Our large number of participating patients, the inclusion of all patients followed-up during the study period regardless of their treatment or final clinical outcome, and the exclusion of patients with missing crucial data may partly overcome the limitations of the design. Third, the possible transmission route was unknown for a substantial number of the study population. Our analysis revealed that the demographic and clinical profile of those patients was similar to that of patients infected through blood transfusions. Therefore, it is most likely, that these patients represent old infections. However, taking into consideration that their date of infection is unknown, this hypothesis cannot be tested. In addition, the genotypes of 5% of our sample were unable to be classified with the present genotyping system and the proportion of non-detectable genotypes remained stable throughout the study period. These may include new subtypes, which may be beyond the scope of the current genotyping method used in this study.

In conclusion, our results show that the epidemiology of HCV infection is changing in southwest China. With the implementation of mandatory HCV screening of blood and blood products, IDU has accounted for the vast majority of all new HCV infections. Due to the rising prevalence of HCV among “new IDUs”, those risk behaviors should become the focus of interventions for the prevention of infectious diseases in the drug using population. Furthermore, there is a need for more systematic treatment of hepatitis C for drug users.
